# Accelerated ^19^F biomolecular magic-angle spinning NMR with paramagnetic dopants

**DOI:** 10.5194/mr-7-29-2026

**Published:** 2026-04-16

**Authors:** Lea M. Becker, Giorgia Toscano, Anna Kapitonova, Rajkumar Singh, Undina Guillerm, Roman J. Lichtenecker, Paul Schanda

**Affiliations:** 1 Institute of Science and Technology Austria, Am Campus 1, 3400 Klosterneuburg, Austria; 2 Institute of Organic Chemistry, University of Vienna, Währinger Str. 38, 1090 Vienna, Austria

## Abstract

The advantageous characteristics attributed to the ${}^{19}F$ nucleus have made it a popular target for nuclear magnetic resonance (NMR) once again in recent years. Aside from solution NMR, an increasing number of studies have been conducted applying solid-state magic-angle spinning (MAS) NMR to fluorine-labelled samples. Here, the high chemical shift anisotropy and strong dipolar couplings can be utilised to get structural insights into proteins and measure long distances. Despite increasing popularity and promising benefits, the sensitivity of biomolecular ${}^{19}F$ MAS NMR often suffers from slow longitudinal $T_{1}$ relaxation and therefore long recycle delays. In this work, we expand paramagnetic doping, an approach commonly used to reduce proton $T_{1}$ relaxation times, to ${}^{19}F$ -labelled biological samples. We study the effect of Gd(DTPA) and Gd(DTPA-BMA) on ${}^{19}F$ $T_{1}$ and $T_{2}$ , and ${}^{13}C$ $T_{1}$ and $T_{2}$ relaxation in a [5- ${}^{19}F^{13}C$ ]-tryptophan-labelled protein via ${}^{19}F$ -detected MAS NMR experiments. The observed paramagnetic relaxation enhancement substantially reduces measurement times of ${}^{19}F$ MAS NMR experiments without compromising resolution. Additionally, we report the chemical shift assignments of all four fluorotryptophan signals in the 12×39 kDa -large protein TET2 using a mutagenesis approach.

## 1 Introduction

Biomolecular ${}^{19}F$ nuclear magnetic resonance (NMR) has regained attention in recent years due to the unique properties of the ${}^{19}F$ nucleus and the diverse labelling strategies for proteins and nucleic acids, which make it a versatile tool for a wide range of applications and systems (Sengupta, 2024; Gronenborn, 2022; Juen et al., 2024; Overbeck et al., 2020; Heller et al., 2024). Recently, there have been exciting developments in the synthesis of compounds for the ${}^{19}F$ -labelling of proteins (Boeszoermenyi et al., 2019; Toscano et al., 2024; Toscano et al., 2024; Suleiman et al., 2024). The introduction of a carbon-13 to form a ${}^{19}F{-}^{13}C$ spin pair facilitates new spectroscopic possibilities such as two-dimensional experiments and the exploration of the ${}^{19}F{-}^{13}C$ TROSY effect. Additional deuteration of labelling compounds can reduce the need for ${}^{1}H$ decoupling and lead to a reduction of unwanted relaxation pathways.

In contrast to solution-state NMR, solid-state magic-angle spinning (MAS) ${}^{19}F$ NMR was challenging for a long time due to the high chemical shift anisotropy (CSA) of the ${}^{19}F$ nucleus and strong dipolar ${}^{1}H{-}^{19}F$ couplings, which can lead to severe line broadening (Ulrich, 2005). Following the development of faster spinning and specialised probe designs that enable efficient averaging and decoupling of the CSA and dipolar couplings, biomolecular ${}^{19}F$ MAS NMR is becoming more feasible. These advancements have led to ${}^{19}F$ NMR studies of protein microcrystals, membrane proteins, and large biomolecular assemblies such as virus capsids. The focus of these studies was set on assignments and structural investigations utilising the possibility of measuring distances of up to 20 Å (Roos et al., 2018; Duan et al., 2022; Shcherbakov et al., 2019; Shcherbakov et al., 2021; Porat-Dahlerbruch et al., 2022; Wang et al., 2018).

Despite the large gyromagnetic ratio of the fluorine nucleus, the sensitivity of ${}^{19}F$ -excited MAS NMR experiments is often limited by long longitudinal $T_{1}$ relaxation times, which are often several seconds (Duan et al., 2022; Roos et al., 2018; Wang et al., 2018; Porat-Dahlerbruch et al., 2022). As the recycle delay $\tau_{r.d.}$ for an optimal signal-to-noise ratio (SNR) is directly related to the longitudinal relaxation of the excited nucleus ( $\tau_{r.d.}^{\mathrm{opt}}=1.26\cdot T_{1}$ ; Schanda, 2009), most of the experiment time is spent waiting for the spin polarisation to build up again. The use of deuterated precursors for ${}^{19}F$ labelling results in even longer $T_{1}$ relaxation, as short-range ${}^{1}H{-}^{19}F$ dipolar couplings are reduced and dipolar relaxation pathways are minimised.

Paramagnetic doping is an established and effective method to reduce the acquisition time of measurements. In solids, it has primarily been used for experiments in which protons are the initially excited nuclei. The addition of a paramagnetic compound to the sample, e.g. ${\mathrm{Cu}}^{2+}$ or ${\mathrm{Gd}}^{3+}$ chelates, results in enhanced nuclear spin relaxation. The longitudinal $\Gamma^{1}$ and transverse $\Gamma^{2}$ paramagnetic relaxation enhancement (PRE) is given by Solomon (1955); Bertini et al. (2001); Bloembergen and Morgan (1961); Konig (1982)

1Γ1≈215μ04π2γI2ge2μB2S(S+1)r6⋅3τc1+ωI2τc2+7τc1+ωe2τc22Γ2≈115μ04π2γI2ge2μB2S(S+1)r6⋅4τc+3τc1+ωI2τc2+13τc1+ωe2τc2,

with the vacuum permeability $\mu_{0}$ , the gyromagnetic ratio of the nucleus $\gamma_{I}$ , the electron g value $g_{e}$ , the Bohr magneton $\mu_{B}$ , the electron spin quantum number S , the electron-nucleus distance r , the rotational correlation time $\tau_{c}$ , and the nuclear and electron Larmor frequencies $\omega_{I}$ and $\omega_{e}$ . The goal is to find a concentration of the paramagnetic compound in the buffer that, on the one hand, significantly accelerates ${}^{1}H$ $T_{1}$ relaxation, allowing a shorter recycle delay and therefore faster acquisition, and, on the other hand, does not shorten $T_{2}$ , so as not to induce line broadening. While the paramagnetic effects depend on the proximity of the unpaired electron, the enhancement of longitudinal relaxation is spread across the molecule from those nuclei directly relaxed by the paramagnetic centre to other nuclei via ${}^{1}H{-}^{1}H$ spin diffusion (Wickramasinghe et al., 2009). The first studies to achieve a significant increase in SNR per unit time utilised ${\mathrm{Cu}}^{2+}$ -EDTA as a dopant, which remains widely used to date (Wickramasinghe et al., 2007; Wickramasinghe et al., 2009). However, the higher PRE effect of ${\mathrm{Gd}}^{3+}$ chelates, such as Gd(DOTA), Gd(DTPA-BMA), or Gd(DTPA), enables the use of lower concentrations of the compound, reducing possible interactions with the studied biomolecule and sample heating (Linser et al., 2007; Ullrich et al., 2014; Mroue et al., 2014; Öster et al., 2019).

Even though paramagnetic doping was mainly applied for its effect on the $T_{1}$ relaxation of protons, it also increases the relaxation of other nuclei, with the strength of the effect being proportional to the squared gyromagnetic ratio of the nucleus (Eq. 1). Due to the high gyromagnetic ratio of ${}^{19}F$ , a significant reduction in measurement time can be expected for biomolecular ${}^{19}F$ MAS NMR experiments with similar concentrations of paramagnetic compounds, as are used for proton-excited experiments.

Transversal and longitudinal PREs of fluorine in solution were measured previously to obtain distance restraints between a fluorine atom and a paramagnetic moiety (Shi et al., 2012; Matei and Gronenborn, 2016; Bondarenko et al., 2019; Huang et al., 2020). In solids, Lu et al. (2020) demonstrated paramagnetic doping with ${\mathrm{Cu}}^{2+}$ ions in the context of fluorinated crystalline pharmaceuticals for structural characterisation by ${}^{19}F$ MAS NMR. They achieved a significant reduction of the ${}^{19}F$ $T_{1}$ relaxation times in their samples, resulting in approximately 2.5 times faster acquisition of fluorine NMR spectra, which highlights the potential benefits of combining paramagnetic doping and ${}^{19}F$ solid-state MAS NMR (Lu et al., 2020).

In this work, we explore the potential benefits of paramagnetic doping for biomolecular ${}^{19}F$ MAS NMR on proteins, which has not been reported so far, to the best of our knowledge. Using the deuterated 5-fluorotryptophan-labelled protein TET2 ( 12×39 kDa ), we measure ${}^{19}F$ $T_{1}$ and $T_{2}$ , and ${}^{13}C$ $T_{1}$ and $T_{2}$ as a function of the concentration of two ${\mathrm{Gd}}^{3+}$ complexes, Gd(DTPA-BMA) and Gd(DTPA). We find that a concentration of 8 mM Gd(DTPA-BMA) is optimal to reduce measurement times of ${}^{19}F$ MAS NMR experiments through a decrease in the ${}^{19}F$ $T_{1}$ relaxation time without significant line broadening.

## 2 Methods

### 2.1 Protein production and purification

The aminopeptidase TET2 from *Pyrococcus horikoshii* (UniProt entry O59196) was produced via overexpression of the pET41c-PhTET2 plasmid in *Escherichia coli* BL21(DE3) RIL cells. The plasmid is available from AddGene (https://www.addgene.org/182428/, last access: 15 February 2026). For the resonance assignment, tryptophans were mutated into phenylalanines in different combinations either as single mutant (mutant 1: W106F) or as triple mutants (mutant 2 (only W106): W136F, W164F, W276F; mutant 3 (only W136): W106F, W164F, W276F; mutant 4 (only W164): W106F, W136F, W276F; mutant 5 (only W276): W106F, W136F, W164F). The ${}^{19}F$ labelling was achieved either with 5-fluoroindole (Sigma-Aldrich, catalogue number F9108) in protonated medium (mutants) or with [ $5{-}^{13}C,3,4,6{-}^{2}H_{3}$ ]-5-fluoroanthranilic acid (5FC-anthranilic acid) in deuterated medium (wild type).

The 5-fluoroanthranilic acid isotopologue was synthesised in-house by adapting the synthetic route reported by Suleiman et al. (2024) to the present labelling scheme; details of the synthesis will be published elsewhere.

The plasmid (kanamycin resistance) was transformed into competent BL21(DE3) RIL cells (chloramphenicol resistance) via heat shock. Unless otherwise mentioned, all cultures contained kanamycin and chloramphenicol, and shaking was performed at 200 rpm and 37 °C.

5-fluoroindole labelling was achieved as follows. After precultures in LB medium and minimal M9 medium, the main culture was inoculated to an optical density at 600 nm (OD_600_) of 0.2 and shaken until it reached 0.6–0.7. At this point, 1 $g\;L^{-1}$ glyphosate (abcr, Karlsruhe, Germany; catalogue number AB505195) was added, and the culture was grown for 15 min before 100 $\mathrm{mg}\;L^{-1}$ 5-fluoroindole, 50 $\mathrm{mg}\;L^{-1}$ *L*-tyrosine (Sigma T3754), and 50 $\mathrm{mg}\;L^{-1}$ *L*-phenylalanine (Sigma 78019) were added. The culture was grown for 45 min , and expression was induced with 1 mM isopropyl- β -D-thiogalactopyranosid (IPTG). Cells were harvested at 6500 rcf for 15 min after 4 h of shaking.

Labelling with 5FC-anthranilic acid was achieved as follows. Cells were adjusted to deuterated M9 medium by growth in consecutive precultures of LB medium and M9 medium prepared with 100 % $H_{2}O$ , 50 % $H_{2}O$ /50 % $D_{2}O$ , and 100 % $D_{2}O$ . The final preculture and the main culture were prepared with ${}^{15}{\mathrm{NH}}_{4}\mathrm{Cl}$ and D- ${}^{2}H_{7}$ -glucose. The main culture was inoculated to an OD_600_ of 0.2 and shaken until it reached 0.6–0.7. At this point, 1 $g\;L^{-1}$ glyphosate, 50 $\mathrm{mg}\;L^{-1}$ *L*-tyrosine, 50 $\mathrm{mg}\;L^{-1}$ *L*-phenylalanine, and 15 $\mathrm{mg}\;L^{-1}$ 5FC-anthranilic acid were added, and the culture was shaken for 40 min . The temperature was then reduced to 28 °C for 15 min before induction with 1 mM IPTG. Cells were grown overnight at 28 °C and then harvested at 6500 rcf for 15 min .

The cell pellet was re-suspended in lysis buffer (50 mM Tris-HCl pH 7.5, 150 mM NaCl, 0.05 $\mathrm{mg}\;{\mathrm{mL}}^{-1}$ DNase, 2 mM ${\mathrm{MgCl}}_{2}$ , 0.025 $\mathrm{mg}\;{\mathrm{mL}}^{-1}$ RNase, and 0.5 tablets cOmplete EDTA-free protease inhibitor), kept on ice for 30 min , and sonicated for 2 min . A heat shock was performed at 80 °C for 15 min . After the addition of 10 mL buffer A (20 mM Tris-HCl pH 7.5, 100 mM NaCl), the cell debris was collected by centrifugation at 46 000 rcf for 40 min at 4 °C. The supernatant was washed with buffer A using an Amicon ultra centrifugal filter with a molecular weight cutoff of 100 kDa before loading onto a RESOURCE Q column (Cytiva) and eluted with a gradient over 10 column volumes from buffer A to buffer B (20 mM Tris-HCl pH 7.5, 1 M NaCl). The fractions containing the protein were concentrated, loaded onto a HiLoad 10/300 Superdex 200 pg column (Cytiva), and eluted in buffer A.

### 2.2 Sample preparation

Samples for solid-state MAS NMR were prepared by batch crystallisation of TET2 with 2-methyl-2,4-pentanediol (MPD; Sigma 68340) (Gauto et al., 2019). A solution of 10 $\mathrm{mg}\;{\mathrm{mL}}^{-1}$ protein and the paramagnetic compound (where applicable; Gd(DTPA): Sigma 381667, Gd(DTPA-BMA): GE Healthcare Omniscan (contains 4.2 % NaCa(DTPA-BMA)) in buffer A was mixed with MPD in a v/v ratio of 1:1 . The concentration given for the paramagnetic compound in the following is related to the final concentration in the sample, including MPD. The microcrystals were filled into a 1.3 mM MAS rotor (Bruker) by ultracentrifugation overnight at 4 °C and 68 000 rcf .

### 2.3 NMR

MAS NMR experiments were performed on a Bruker Avance Neo spectrometer operating at 14.09 T (600 MHz ${}^{1}H$ Larmor frequency). A triple-resonance HFX probe head from PhoenixNMR equipped with a 1.3 mm MAS stator from Bruker was used, with the X channel tuned to ${}^{13}C$ . Temperature calibration was done with an external ${}^{2}H_{4}$ -methanol sample (Karschin et al., 2022), and chemical shift referencing was done indirectly via the ${}^{1}H$ signal of 2,2-dimethyl-2-silapentane-5-sulfonate sodium salt (DSS). All experiments were performed at a MAS frequency of 55.555 kHz and a sample temperature of approximately 309 K . Spectra were processed with Bruker Topspin software (versions 4.1.4 and 4.5.0).

**Figure 1:**
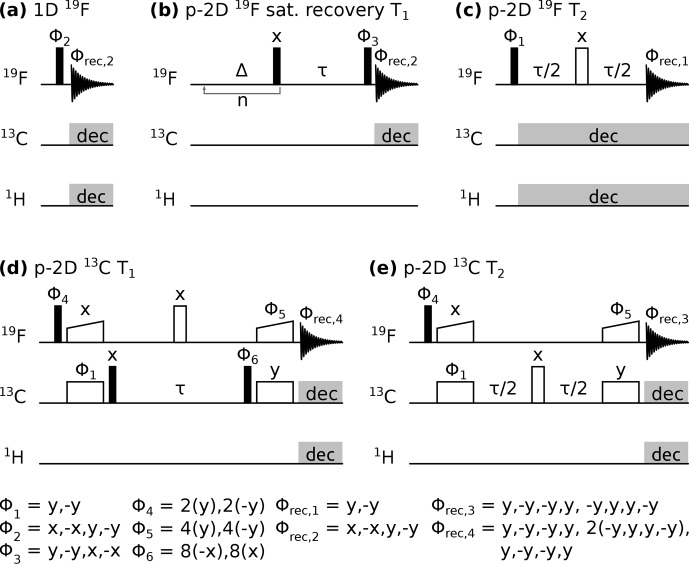
Pulse sequences used in this study. Closed, open, and wide-open rectangles denote 90 °, 180° , and CP spin-lock pulses, respectively. Grey rectangles indicate composite pulse decoupling. Δ and τ are delays, and n indicates a loop. Pulse phases are indicated above the pulse, with $\Phi_{n}$ marking a pulse undergoing phase cycling, as noted below. Acquisition is denoted with a free induction decay scheme with the receiver phase $\Phi_{\mathrm{rec},n}$ indicated above. p-2D stands for pseudo two-dimensional spectrum with one frequency dimension and one pseudo dimension in which a delay τ is incremented.

Pulse sequence diagrams can be found in Fig. 1. All experiments were performed with ${}^{19}F$ detection (10 ms acquisition time), and ${}^{1}H$ and ${}^{13}C$ decoupling unless stated otherwise. Composite pulse decoupling during acquisition was typically achieved with 10 kHz swfTPPM (Thakur et al., 2006) on ${}^{1}H$ and 10 kHz WALTZ-16 (Shaka et al., 1983) on ${}^{13}C$ . The recycle delay was set to $\approx 1.3\cdot T_{1}$ of ${}^{19}F$ , depending on the concentration of the paramagnetic compound, except for the ${}^{19}F$ saturation recovery experiment, in which the recycle delay was set to 1.2 s . The pre-saturation block in the saturation recovery experiment was repeated n=50 times with a delay Δ=4.5ms . Magnetisation transfer in ${}^{13}C$ relaxation experiments was achieved via dipolar ${}^{19}F{-}^{13}C$ cross-polarisation (CP) steps. Typical CP spin-lock radio-frequency field strengths were 40 kHz on ${}^{13}C$ and 90 kHz on ${}^{19}F$ , with a linear ramp of 90 %–100 % and a transfer time of 400 µs . ${}^{19}F$ and ${}^{13}C$ relaxation experiments were recorded as pseudo two-dimensional spectra with one ${}^{19}F$ frequency dimension and one pseudo dimension in which a relaxation delay τ was incremented.

### 2.4 Relaxation rate analysis

Spectra were processed as pseudo two-dimensional spectra in Topspin and converted to UCSF format with the bruk2ucsf program provided in Sparky (Goddard and Kneller, 2008). Python scripts for analysis of relaxation rate constants were written in-house, utilising the Nmrglue package (Helmus and Jaroniec, 2013). Spectra were split into individual 1D slices, and peaks were fitted with the routine implemented in Nmrglue. The intensities were then fitted to a mono-exponential function. Errors were determined by Monte Carlo analysis (500 iterations) using one standard deviation of the spectral noise.

## 3 Results and discussion

### 3.1 Assignment of fluorine-labelled tryptophans in TET2

**Figure 2:**
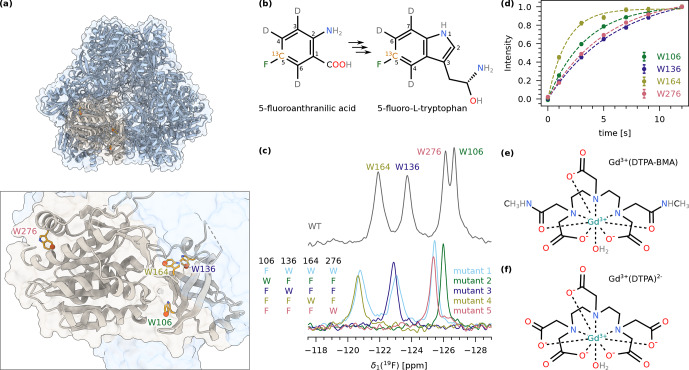
**(a)** Structure of the dodecameric TET2 (PDB: 1Y0R) (Borissenko and Groll, 2005) in cartoon representation with one subunit highlighted in beige (upper panel). The lower panel shows a close-up of one subunit with the four tryptophans indicated as orange sticks. Position 5 in the tryptophan ring is highlighted with spheres. **(b)** Structure of 5-fluoroanthranilic acid (left), which is converted into 5-fluoro-*L*-tryptophan (right) by the bacteria. **(c)** 1D ${}^{19}F$ MAS NMR spectrum of 5FC-W-TET. The upper panel displays the spectrum of the wild type, showing all four tryptophan peaks. The lower panel shows spectra of the five assignment mutants (mutant 1: W106F; mutant 2: W136F, W164F, W276F; mutant 3: W106F, W164F, W276F; mutant 4: W106F, W136F, W276F; mutant 5: W106F, W136F, W164F; see the Methods section for details). The resulting assignment is indicated at the top. The wild-type spectrum is shifted due to the isotope shift, as this sample is deuterated compared to the mutants. **(d)** Exponential fits of ${}^{19}F$ saturation recovery experiments on 5FC-W-TET without paramagnetic dopant. **(e)** Structure of Gd(DTPA-BMA). **(f)** Structure of Gd(DTPA).

TET2 is a dodecameric aminopeptidase from *P. horikoshii* that has been studied extensively by MAS NMR previously (Gauto et al., 2019; Gauto et al., 2022). Each of the 12 identical subunits contains four tryptophan residues: W106, W136, W164, and W276 (Fig. 2a). To achieve fluorine labelling of the aromatic ring, we expressed TET2 in deuterated M9 medium and added 5-fluoroanthranilic acid, which is metabolised by the bacteria into 5-fluoro-*L*-tryptophan (Fig. 2b). This precursor has a ${}^{19}F{-}^{13}C$ spin pair at position 5 in the aromatic ring and is deuterated at positions 3, 4, and 6. We will refer to the labelled protein as 5FC-W-TET in the remainder of this discussion. As expected, the ${}^{19}F$ MAS NMR spectrum of 5FC-W-TET shows four individual peaks (Fig. 2c, upper spectrum). For the assignment of the signals, we prepared five Trp to Phe mutants in non-deuterated medium using the commercially available precursor 5-fluoroindole (see the Methods section for details). The respective spectra show either one (triple mutants) or three (single mutant) signals, which allowed us to assign the four signals (Fig. 2c, bottom spectra). Note that the spectrum of the deuterated wild type is shifted due to an isotope shift (Luck et al., 1996).

### 3.2 The effect of Gd(DTPA-BMA) and Gd(DTPA) on bulk ${}^{19}F$ and ${}^{13}C$ relaxation

In recent years, several studies have been published using biomolecular ${}^{19}F$ MAS NMR to study structural aspects of proteins (Roos et al., 2018; Duan et al., 2022; Shcherbakov et al., 2019; Shcherbakov et al., 2021; Porat-Dahlerbruch et al., 2022; Wang et al., 2018). The ${}^{19}F$ $T_{1}$ relaxation time was often reported to be several seconds long, leading to a long $\tau_{r.d.}^{\mathrm{opt}}$ to obtain an optimal SNR. We measured ${}^{19}F$ $T_{1}$ of 5FC-W-TET with a saturation recovery experiment (Fig. 2d), and obtained values between 1.67±0.08s (W164) and 6.0±0.4s (W136). Considering the highest value, this would correspond to a recycle delay of $\tau_{r.d.}^{\mathrm{opt}}=7.56\;s$ .

**Figure 3:**
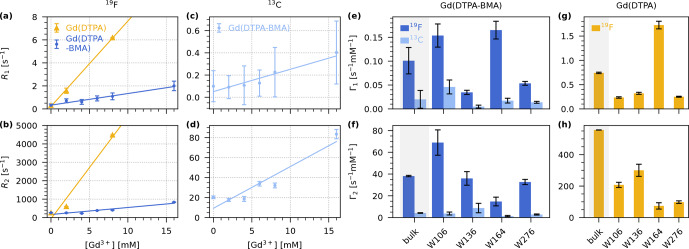
${}^{19}F$ and ${}^{13}C$ longitudinal and transverse PRE ( $\Gamma_{1}$ and $\Gamma_{2}$ ) for Gd(DTPA-BMA) and Gd(DTPA) measured on 5FC-W-Trp. **(a–b)** Linear fits of bulk ${}^{19}F$ $R_{1}$ **(a)** and $R_{2}$ **(b)** relaxation rate constants as a function of the concentration of Gd(DTPA-BMA) (blue) and Gd(DTPA) (yellow). **(c–d)** Linear fits of bulk ${}^{13}C$ $R_{1}$ **(c)** and $R_{2}$ **(d)** relaxation rate constants as a function of the concentration of Gd(DTPA-BMA) (light blue). **(e–f)** ${}^{19}F$ (dark blue) and ${}^{13}C$ (light blue) $\Gamma_{1}$ **(e)** and $\Gamma_{2}$ **(f)** for Gd(DTPA-BMA). **(g–h)** ${}^{19}F$ $\Gamma_{1}$ **(g)** and $\Gamma_{2}$ **(h)** for Gd(DTPA). The bulk relaxation rate constants in **(a)**–**(d)** are the average over the four residues (see Figs. S1, S2, S3, S4, S5) except for the rate at 8 mM Gd(DTPA), which was only measured as a bulk rate due to line broadening. In **(e)**–**(h)**, the first bar of each plot is the bulk PRE resulting from the fits in **(a)**–**(d)** (grey background), followed by the individual values for each residue resulting from the fits in Figs. S1, S2, S3, S4, S5. Note that the per-residue fits for Gd(DTPA) **(g, h)** are performed with only two points (0 and 2 mM ), while the bulk fit is performed with three points (0, 2, and 8 mM ). This leads to a deviation between $\Gamma^{\mathrm{bulk}}$ and the average over the residue-wise values.

To reduce the ${}^{19}F$ $T_{1}$ , we used paramagnetic doping with two different ${\mathrm{Gd}}^{3+}$ chelates. We prepared samples with six concentrations of Gd(DTPA-BMA) (0, 2, 4, 6, 8, and 16 mM , Fig. 2e), and measured ${}^{19}F$ $T_{1}$ and $T_{2}$ , and ${}^{13}C$ $T_{1}$ and $T_{2}$ for each residue (Figs. S1, S2, S3, S4, S6 in the Supplement). For Gd(DTPA) (Fig. 2f), we prepared samples with 2 and 8 mM of the compound and measured ${}^{19}F$ $T_{1}$ and $T_{2}$ (Fig. S5, S6). The bulk relaxation rates $R=T^{-1}$ (average over all residues) are shown in Fig. 3a–d.

At 8 mM Gd(DTPA), the increase in $R_{2}$ led to a broadening of the whole spectrum that made the individual peaks indistinguishable (Fig. S7). Additionally, Gd(DTPA) changed the crystallisation behaviour of TET2, possibly due to binding to the protein surface (Fig. S8) (Petros et al., 1990). We therefore refrained from preparing samples with other concentrations or measuring ${}^{13}C$ relaxation rate constants.

We find that both compounds lead to an increase in $R_{1}$ as well as $R_{2}$ . To quantify the effect of the two compounds, we determined the longitudinal and transverse PREs ( $\Gamma_{1}$ and $\Gamma_{2}$ ), which are given by the slope of a linear fit of the respective relaxation rate constants as a function of the concentration of paramagnetic dopant. We performed fits of the bulk and per-residue relaxation rate constants to obtain the PREs for Gd(DTPA-BMA) ( ${}^{19}F$ and ${}^{13}C$ ; Fig. 3e–f) and for Gd(DTPA) (only ${}^{19}F$ ; Fig. 3g–h).

As expected, both $\Gamma_{1}$ and $\Gamma_{2}$ are smaller for ${}^{13}C$ than for ${}^{19}F$ . This is due to the smaller gyromagnetic ratio of the carbon nucleus (Eq. 1).

Interestingly, the measured ${}^{19}F$ PREs for Gd(DTPA) are much higher than for Gd(DTPA-BMA). The bulk effect of Gd(DTPA) on $R_{1}$ ( $\Gamma_{1}=0.743\pm 0.015\;s^{-1}\;{\mathrm{mM}}^{-1}$ ) is roughly seven times stronger than the effect of Gd(DTPA-BMA) ( $\Gamma_{1}=0.101\pm 0.028\;s^{-1}\;{\mathrm{mM}}^{-1}$ ). For $\Gamma_{2}$ , the difference is even bigger: The bulk value for Gd(DTPA) ( $\Gamma_{2}=556.2\pm 0.7\;s^{-1}\;{\mathrm{mM}}^{-1}$ ) is over 14 times higher than the value for Gd(DTPA-BMA) ( $\Gamma_{2}=38.2\pm 0.6\;s^{-1}\;{\mathrm{mM}}^{-1}$ ). The differences in relaxation behaviour are likely the result of the specific physicochemical properties of the two compounds (e.g. the slower water exchange rate of Gd(DTPA-BMA)) (Caravan et al., 1999).

**Figure 4:**
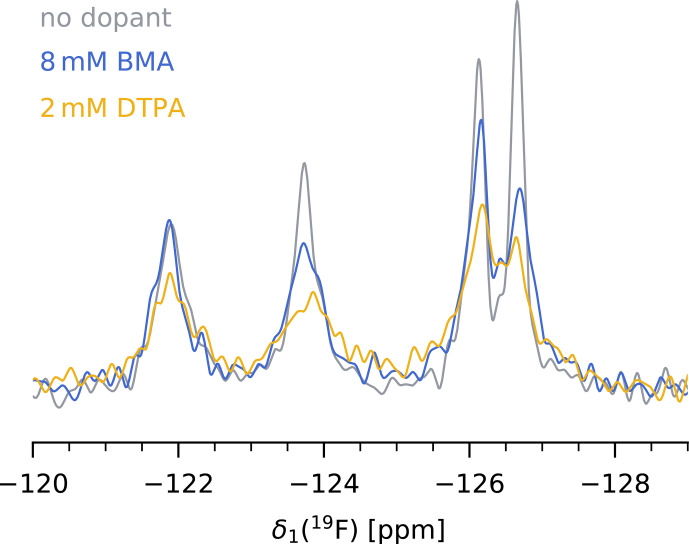
${}^{19}F$ MAS NMR spectrum of 5FC-W-TET without dopant (grey), with 8 mM Gd(DTPA-BMA) (blue), and with 2 mM Gd(DTPA) (yellow). $\tau_{r.d.}$ was set to five times the ${}^{19}F$ $T_{1}$ of the slowest relaxing peak for each sample. Note that the absolute intensities between samples are not comparable, as the amount of protein inside the rotor is hard to determine.

We find that in our case, paramagnetic doping with 8 mM Gd(DTPA-BMA) is the best compromise between a significant reduction of ${}^{19}F$ $T_{1}$ without heavily compromising $T_{2}$ and therefore spectral resolution. The decrease in bulk $T_{1}$ from 3.2±1.3s at 0 mM to 0.92±0.25s at 8 mM Gd(DTPA-BMA) translates to a reduction of $\tau_{r.d.}^{\mathrm{opt}}$ from 4.0±1.6 to 1.2±0.4s . The more than 3-fold shorter recycle delay significantly reduces the measurement times of ${}^{19}F$ MAS NMR spectra, or, in other words, increases the SNR per unit time. Although the same effect could be achieved with lower concentrations of Gd(DTPA), we prefer the use of Gd(DTPA-BMA) due to a potential interaction of Gd(DTPA) with the protein in our case (see above). Figure 4 shows a comparison of the ${}^{19}F$ spectra of 5FC-W-TET with and without dopant ( ${}^{13}C$ spectra are shown in Fig. S9).

### 3.3 The relative PREs of individual tryptophan residues

The observed PREs for the individual tryptophans differ (Fig. 3e–h), which can be expected as they are impacted by a multitude of factors, such as the solvent accessibility of the residue, its dynamics, or the density of surrounding protons (Tang et al., 2011; Wickramasinghe et al., 2009). The influences of these factors can conversely even be used to characterise the surfaces and interaction interfaces of proteins with so-called solvent PREs (Pintacuda and Otting, 2002; Öster et al., 2017; Hocking et al., 2013).

To rationalise the observed PRE data for the four Trp sites, we compared them to structural parameters. We reasoned that the relaxation properties may be impacted by the ${}^{1}H$ spins surrounding each of the ${}^{19}F$ Trp sites and calculated the root-sum-square dipolar coupling $d^{\mathrm{rss}}$ (Fig. S10a). This parameter approximates the effective dipolar coupling network and can serve as an indicator for spin diffusion, which influences the propagation of the PRE effect throughout the protein. It was calculated as the square root of the sum of squared dipolar couplings between a fluorine and all back-exchangeable protons (Zorin et al., 2006). Moreover, we calculated the solvent accessible surface area (SASA) of each tryptophan (Fig. S10b), which is an approximate measure of the shortest distance between the paramagnetic compound and a given ${}^{19}F$ atom. In our case, we did not find a direct correlation of these parameters with the observed PREs (Fig. S10c–d).

Interestingly, the PRE patterns (relative strength of the measured PREs for the four residues) are different for different relaxation rate constants ( $\Gamma_{1}$ and $\Gamma_{2}$ ), different compounds, and different nuclei ( ${}^{19}F$ and ${}^{13}C$ ) (Fig. 3e–h). In addition to the factors mentioned above, other influencing parameters that could explain these different patterns include a lower spin diffusion efficiency for ${}^{13}C$ , specific binding of the ${\mathrm{Gd}}^{3+}$ complex to the protein, and properties of the compound, such as the rotational correlation time $\tau_{c}$ or the water exchange rate.

Differences between the patterns observed for Gd(DTPA-BMA) and Gd(DTPA) could be due to the specific binding of one of the complexes to the protein. A decrease in the distance r between the compound and residues close to the binding site would increase the observed PRE effects as both $\Gamma_{1}$ and $\Gamma_{2}$ are proportional to $r^{-6}$ (Eq. 1). The binding would also decrease the $\tau_{c}$ of the compound, which could, in combination with the dynamics of a specific tryptophan, lead to dampening or acceleration of the PRE.

The most striking observation is that the ${}^{19}F$ $\Gamma^{1}$ of W164 is very high relative to the other residues compared to ${}^{19}F$ $\Gamma^{2}$ for both ${\mathrm{Gd}}^{3+}$ complexes. Such a difference between the longitudinal and transverse PRE can also be a result of a local reduction of $\tau_{c}$ due to binding. As $\Gamma_{1}$ and $\Gamma_{2}$ sample the spectral density at different frequencies, their reaction to changes of $\tau_{c}$ is not the same (Eq. 1) (Jaroniec, 2012). The complex interplay of diverse parameters makes it difficult to understand these patterns in detail.

## 4 Conclusions

The potential of biomolecular ${}^{19}F$ MAS NMR is often limited by long $T_{1}$ relaxation times that require recycle delays of several seconds. In this work, we discussed the application of paramagnetic doping with ${\mathrm{Gd}}^{3+}$ complexes to accelerate these experiments. Previously, paramagnetic doping with different compounds was used to reduce the ${}^{1}H$ $T_{1}$ in a variety of sample types, such as membrane proteins and protein microcrystals (Wickramasinghe et al., 2007; Wickramasinghe et al., 2009; Linser et al., 2007; Ullrich et al., 2014). To our knowledge, this is the first study applying paramagnetic doping for MAS NMR to fluorine-labelled biological samples.

We evaluated the effect of two different ${\mathrm{Gd}}^{3+}$ complexes – Gd(DTPA-BMA) and Gd(DTPA) – on the ${}^{19}F$ $T_{1}$ and $T_{2}$ , and ${}^{13}C$ $T_{1}$ and $T_{2}$ relaxation times in deuterated and 5-fluorotryptophan-labelled TET2. The addition of 8 mM Gd(DTPA-BMA) reduces $\tau_{r.d.}^{\mathrm{opt}}$ by a factor of more than 3 compared to the undoped sample, without causing significant line broadening. The addition of Gd(DTPA) results in a stronger paramagnetic relaxation enhancement, but it is a less favourable compound due to its interaction with the protein.

We anticipate that the use of paramagnetic doping for biomolecular ${}^{19}F$ MAS NMR can be applied to a variety of systems, experiments, and different types of sample preparations. The increase in sensitivity will be especially beneficial for structural studies and the measurement of anisotropic spin interactions. As for paramagnetic doping of non-fluorine-labelled samples, the optimal concentration and compound are likely to depend on the specific experimental setup.

## Supplement

10.5194/mr-7-29-2026-supplement The supplement related to this article is available online at https://doi.org/10.5194/mr-7-29-2026-supplement.

## Data Availability

NMR spectra, analysis scripts, and raw data are publicly available at the Institute of Science and Technology Austria (ISTA) research explorer (10.15479/AT-ISTA-21284; Becker and Schanda, 2026).

## References

[bib1.bibx1] Becker LM, Schanda P (2026). Institute of Science and Technology Austria [data set].

[bib1.bibx2] Bertini I, Luchinat C, Giacomo P (2001). Solution NMR of Paramagnetic Molecules: Applications to Metallobiomolecules and Models.

[bib1.bibx3] Bloembergen N, Morgan LO (1961). Proton relaxation times in paramagnetic solutions. Effects of electron spin relaxation. J Chem Phys.

[bib1.bibx4] Boeszoermenyi A, Chhabra S, Dubey A, Radeva DL, Burdzhiev NT, Chanev CD, Petrov OI, Gelev VM, Zhang M, Anklin C, Kovacs H, Wagner G, Kuprov I, Takeuchi K, Arthanari H (2019). Aromatic ^19^F-^13^C TROSY: a background-free approach to probe biomolecular structure, function, and dynamics. Nat Methods.

[bib1.bibx5] Bondarenko V, Wells MM, Chen Q, Singewald KC, Saxena S, Xu Y, Tang P (2019). ^19^F Paramagnetic Relaxation-Based NMR for Quaternary Structural Restraints of Ion Channels. ACS Chem Biol.

[bib1.bibx6] Borissenko L, Groll M (2005). Crystal structure of TET protease reveals complementary protein degradation pathways in prokaryotes. J Mol Biol.

[bib1.bibx7] Caravan P, Ellison JJ, McMurry TJ, Lauffer RB (1999). Gadolinium(III) chelates as MRI contrast agents: Structure, dynamics, and applications. Chem Rev.

[bib1.bibx8] Duan P, Dregni AJ, Hong M (2022). Solid-State NMR ^19^F-^1^H-^15^N Correlation Experiments for Resonance Assignment and Distance Measurements of Multifluorinated Proteins. J Phys Chem A.

[bib1.bibx9] Gauto DF, Estrozi LF, Schwieters CD, Effantin G, Macek P, Sounier R, Sivertsen AC, Schmidt E, Kerfah R, Mas G, Colletier JP, Güntert P, Favier A, Schoehn G, Schanda P, Boisbouvier J (2019). Integrated NMR and cryo-EM atomic-resolution structure determination of a half-megadalton enzyme complex. Nat Commun.

[bib1.bibx10] Gauto DF, Macek P, Barducci A, Fraga H, Hessel A, Terauchi T, Gajan D, Miyanoiri Y, Boisbouvier J, Lichtenecker R, Kainosho M, Schanda P (2019). Aromatic Ring Dynamics, Thermal Activation, and Transient Conformations of a 468 kDa Enzyme by Specific ^1^H-^13^C Labeling and Fast Magic-Angle Spinning NMR. J Am Chem Soc.

[bib1.bibx11] Gauto DF, Macek P, Malinverni D, Fraga H, Paloni M, Sučec I, Hessel A, Bustamante JP, Barducci A, Schanda P (2022). Functional control of a 0.5 MDa TET aminopeptidase by a flexible loop revealed by MAS NMR. Nat Commun.

[bib1.bibx12] Goddard TD, Kneller DG (2008). SPARKY 3.

[bib1.bibx13] Gronenborn AM (2022). Small, but powerful and attractive: ^19^F in biomolecular NMR. Structure.

[bib1.bibx14] Heller GT, Shukla VK, Figueiredo AM, Hansen DF (2024). Picosecond Dynamics of a Small Molecule in Its Bound State with an Intrinsically Disordered Protein. J Am Chem Soc.

[bib1.bibx15] Helmus JJ, Jaroniec CP (2013). Nmrglue: An open source Python package for the analysis of multidimensional NMR data. J Biomol NMR.

[bib1.bibx16] Hocking HG, Zangger K, Madl T (2013). Studying the structure and dynamics of biomolecules by using soluble paramagnetic probes. Chem Phys Chem.

[bib1.bibx17] Huang Y, Wang X, Lv G, Razavi AM, Huysmans GH, Weinstein H, Bracken C, Eliezer D, Boudker O (2020). Use of paramagnetic ^19^F NMR to monitor domain movement in a glutamate transporter homolog. Nat Chem Biol.

[bib1.bibx18] Jaroniec CP (2012). Solid-state nuclear magnetic resonance structural studies of proteins using paramagnetic probes. Solid State Nucl Magn Reson.

[bib1.bibx19] Juen F, Glänzer D, Plangger R, Kugler V, Fleischmann J, Stefan E, Case DA, Kovacs H, Dayie TK, Kreutz C (2024). Enhanced TROSY Effect in [2-^19^F, 2-^13^C] Adenosine and ATP Analogs Facilitates NMR Spectroscopy of Very Large Biological RNAs in Solution. Angew Chem Int Ed.

[bib1.bibx20] Karschin N, Krenek S, Heyer D, Griesinger C (2022). Extension and improvement of the methanol-d4 NMR thermometer calibration. Magn Reson Chem.

[bib1.bibx21] Konig SH (1982). A classical description of relaxation of interacting pairs of unlike spins: Extension to 
$T_{1\rho }$


$T_{2}$
, and 
$T_{1\rho \mathrm{off}}$
, including contact interactions. J Magn Reson.

[bib1.bibx22] Linser R, Chevelkov V, Diehl A, Reif B (2007). Sensitivity enhancement using paramagnetic relaxation in MAS solid-state NMR of perdeuterated proteins. J Magn Reson.

[bib1.bibx23] Lu X, Lu X, Tsutsumi Y, Huang C, Xu W, Byrn SR, Templeton AC, Buevich AV, Amoureux JP, Amoureux JP, Amoureux JP, Su Y, Su Y, Su Y (2020). Molecular packing of pharmaceuticals analyzed with paramagnetic relaxation enhancement and ultrafast magic angle pinning NMR. Phys Chem Chem Phys.

[bib1.bibx24] Luck LA, Vance JE, O'Connell TM, London RE (1996). ^19^F NMR relaxation studies on 5-fluorotryptophan- and tetradeutero-5-fluorotryptophan-labeled E. coli Glucose/Galactose Receptor. J Biomol NMR.

[bib1.bibx25] Matei E, Gronenborn AM (2016). ^19^F Paramagnetic Relaxation Enhancement: A Valuable Tool for Distance Measurements in Proteins. Angew Chem Int Ed.

[bib1.bibx26] Mroue KH, Zhang R, Zhu P, McNerny E, Kohn DH, Morris MD, Ramamoorthy A (2014). Acceleration of natural-abundance solid-state MAS NMR measurements on bone by paramagnetic relaxation from gadolinium-DTPA. J Magn Reson.

[bib1.bibx27] Öster C, Kosol S, Hartlmüller C, Lamley JM, Iuga D, Oss A, Org ML, Vanatalu K, Samoson A, Madl T, Lewandowski JR (2017). Characterization of Protein-Protein Interfaces in Large Complexes by Solid-State NMR Solvent Paramagnetic Relaxation Enhancements. J Am Chem Soc.

[bib1.bibx28] Öster C, Kosol S, Lewandowski JR (2019). Quantifying Microsecond Exchange in Large Protein Complexes with Accelerated Relaxation Dispersion Experiments in the Solid State. Sci Rep.

[bib1.bibx29] Overbeck JH, Kremer W, Sprangers R (2020). A suite of ^19^F based relaxation dispersion experiments to assess biomolecular motions. J Biomol NMR.

[bib1.bibx30] Petros AM, Mueller L, Kopple KD (1990). NMR Identification of Protein Surfaces Using Paramagnetic Probes. Biochemistry.

[bib1.bibx31] Pintacuda G, Otting G (2002). Identification of protein surfaces by NMR measurements with a paramagnetic Gd(III) chelate. J Am Chem Soc.

[bib1.bibx32] Porat-Dahlerbruch G, Struppe J, Quinn CM, Gronenborn AM, Polenova T (2022). Determination of accurate ^19^F chemical shift tensors with R-symmetry recoupling at high MAS frequencies (60–100 kHz). J Magn Reson.

[bib1.bibx33] Roos M, Wang T, Shcherbakov AA, Hong M (2018). Fast Magic-Angle-Spinning ^19^F Spin Exchange NMR for Determining Nanometer ^19^F-^19^F Distances in Proteins and Pharmaceutical Compounds. J Phys Chem B.

[bib1.bibx34] Schanda P (2009). Fast-pulsing longitudinal relaxation optimized techniques: Enriching the toolbox of fast biomolecular NMR spectroscopy. Progr Nucl Magn Reson Spectrosc.

[bib1.bibx35] Sengupta I (2024). Insights into the Structure and Dynamics of Proteins from ^19^F Solution NMR Spectroscopy. Biochemistry.

[bib1.bibx36] Shaka AJ, Keeler J, Frenkiel T, Freeman R (1983). An improved sequence for broadband decoupling: WALTZ-16. J Magn Reson.

[bib1.bibx37] Shcherbakov AA, Mandala VS, Hong M (2019). High-Sensitivity Detection of Nanometer ^1^H-^19^F Distances for Protein Structure Determination by ^1^H-Detected Fast MAS NMR. J Phys Chem B.

[bib1.bibx38] Shcherbakov AA, Hisao G, Mandala VS, Thomas NE, Soltani M, Salter EA, Davis JH, Henzler-Wildman KA, Hong M (2021). Structure and dynamics of the drug-bound bacterial transporter EmrE in lipid bilayers. Nat Commun.

[bib1.bibx39] Shi P, Li D, Li J, Chen H, Wu F, Xiong Y, Tian C (2012). Application of site-specific ^19^F paramagnetic relaxation enhancement to distinguish two different conformations of a multidomain protein. J Phys Chem Lett.

[bib1.bibx40] Solomon I (1955). Relaxation processes in a system of two spins. Phys Rev.

[bib1.bibx41] Suleiman M, Frere GA, Törner R, Tabunar L, Bhole GV, Taverner K, Tsuchimura N, Pichugin D, Lichtenecker RJ, Vozny O, Gunning P, Arthanari H, Sljoka A, Prosser RS (2024). Characterization of conformational states of the homodimeric enzyme fluoroacetate dehalogenase by ^19^F-^13^C two-dimensional NMR. RSC Chem Biol.

[bib1.bibx42] Tang M, Berthold DA, Rienstra CM (2011). Solid-state NMR of a large membrane protein by paramagnetic relaxation enhancement. J Phys Chem Lett.

[bib1.bibx43] Thakur RS, Kurur ND, Madhu PK (2006). Swept-frequency two-pulse phase modulation for heteronuclear dipolar decoupling in solid-state NMR. Chem Phys Lett.

[bib1.bibx44] Toscano G, Holzinger J, Nagl B, Kontaxis G, Kählig H, Konrat R, Lichtenecker RJ (2024). Decorating phenylalanine side-chains with triple labeled ^13^C/^19^F/^2^H isotope patterns. J Biomol NMR.

[bib1.bibx45] Toscano G, Rosati M, Barbieri L, Maier K, Banci L, Luchinat E, Konrat R, Lichtenecker RJ (2024). The synthesis of specifically isotope labelled fluorotryptophan and its use in mammalian cell-based protein expression for ^19^F-NMR applications. Chem Commun.

[bib1.bibx46] Ullrich SJ, Hölper S, Glaubitz C (2014). Paramagnetic doping of a 7TM membrane protein in lipid bilayers by Gd 3+-complexes for solid-state NMR spectroscopy. J Biomol NMR.

[bib1.bibx47] Ulrich AS (2005). Solid state ^19^F NMR methods for studying biomembranes. Prog Nucl Magn Reson Spectrosc.

[bib1.bibx48] Wang M, Lu M, Fritz MP, Quinn CM, Byeon IJL, Byeon CH, Struppe J, Maas W, Gronenborn AM, Polenova T (2018). Fast Magic-Angle Spinning ^19^F NMR Spectroscopy of HIV-1 Capsid Protein Assemblies. Angew Chem Int Ed.

[bib1.bibx49] Wickramasinghe NP, Kotecha M, Samoson A, Past J, Ishii Y (2007). Sensitivity enhancement in ^13^C solid-state NMR of protein microcrystals by use of paramagnetic metal ions for optimizing ^1^H 
$T_{1}$
 relaxation. J Magn Reson.

[bib1.bibx50] Wickramasinghe NP, Parthasarathy S, Jones CR, Bhardwaj C, Long F, Kotecha M, Mehboob S, Fung LW, Past J, Samoson A, Ishii Y (2009). Nanomole-scale protein solid-state NMR by breaking intrinsic ^1^H 
$T_{1}$
 boundaries. Nat Methods.

[bib1.bibx51] Zorin VE, Brown SP, Hodgkinson P (2006). Quantification of homonuclear dipolar coupling networks from magic-angle spinning ^1^H NMR. Mol Phys.

