# Supplementary material for: Accelerated 19F biomolecular magic-angle spinning NMR with paramagnetic dopants
Source: Magn Reson (Gott). 2026 Apr 16;7(1):29–37. doi: 10.5194/mr-7-29-2026 (PMC13123408; doi:10.5194/mr-7-29-2026)
Supplement: The supplement related to this article is available online at https://doi.org/10.5194/mr-7-29-2026-supplement. [file mr-7-29-2026-supplement.pdf]

Supplement of Magn. Reson., 7, 29–37, 2026
https://doi.org/10.5194/mr-7-29-2026-supplement
© Author(s) 2026. CC BY 4.0 License.

Open Access
MAGNETIC RESONANCE

Supplement of

Accelerated ^{19}F biomolecular magic-angle spinning NMR with paramagnetic dopants

Lea M. Becker et al.

Correspondence to: Paul Schanda (paul.schanda@ist.ac.at)

The copyright of individual parts of the supplement might differ from the article licence.

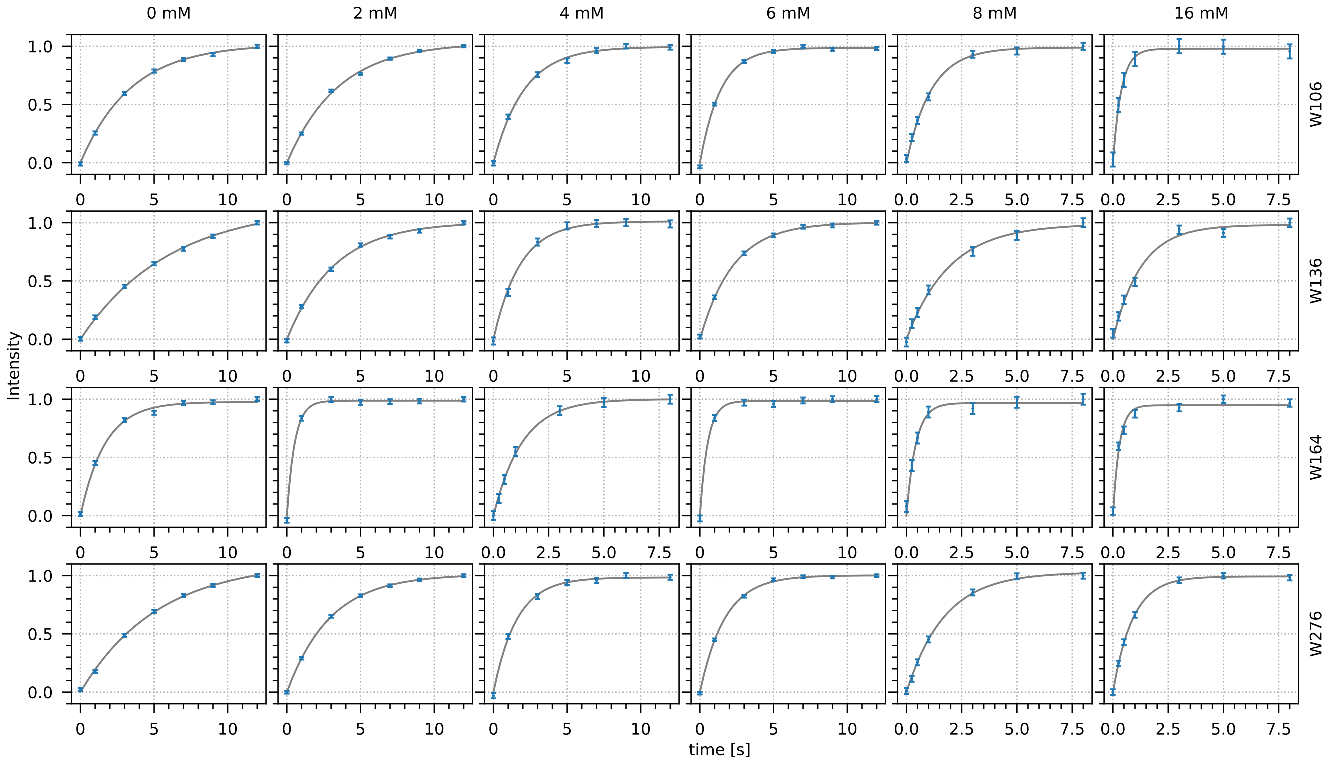

Figure S1: Residue-wise exponential fits of ^{19}F R_1 saturation-recovery relaxation experiments (Fig. 1b) at seven different concentrations of Gd(DTPA-BMA). Concentrations and residues are indicated on the top and at the right side, respectively.

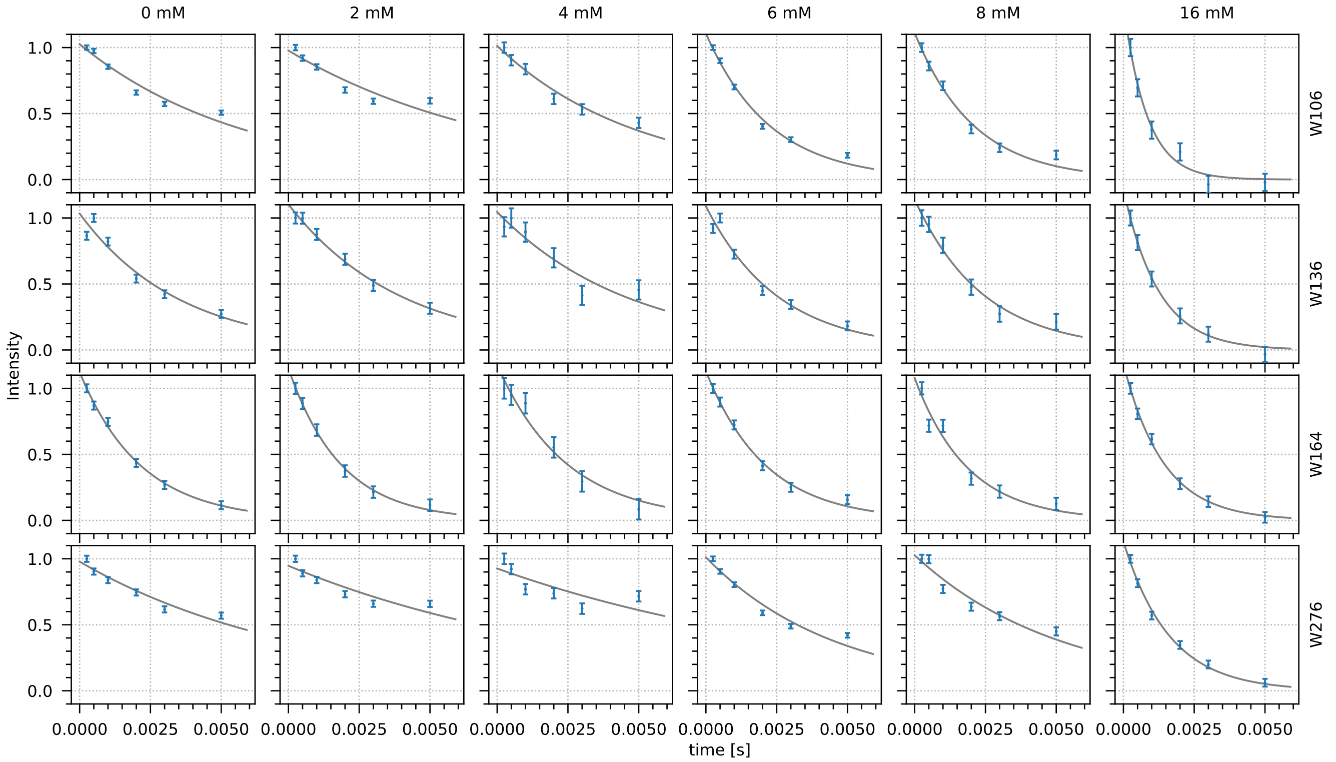

Figure S2: Residue-wise exponential fits of ^{19}F R_2 relaxation experiments (Fig. 1c) at seven different concentrations of Gd(DTPA-BMA). Concentrations and residues are indicated on the top and at the right side, respectively.

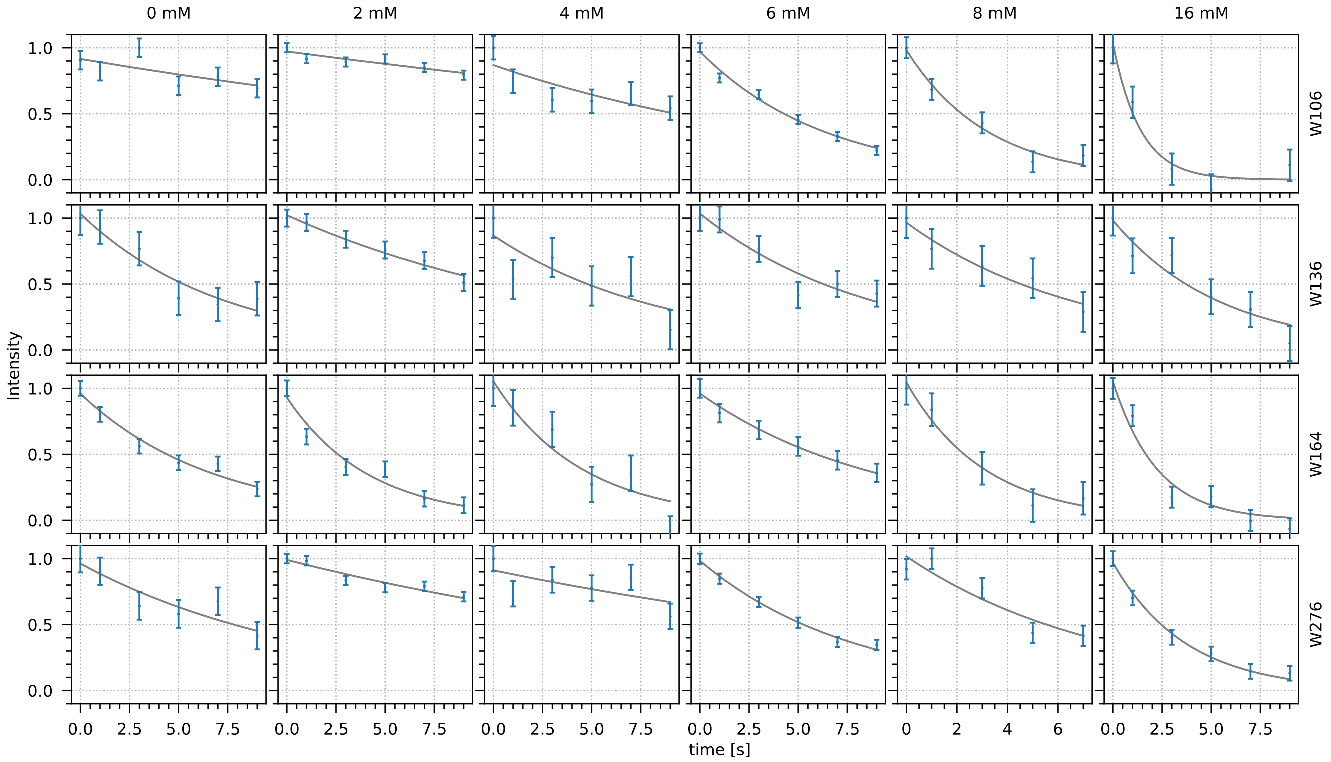

Figure S3: Residue-wise exponential fits of ^{13}C R_1 relaxation experiments (Fig. 1d) at seven different concentrations of Gd(DTPA-BMA). Concentrations and residues are indicated on the top and at the right side, respectively.

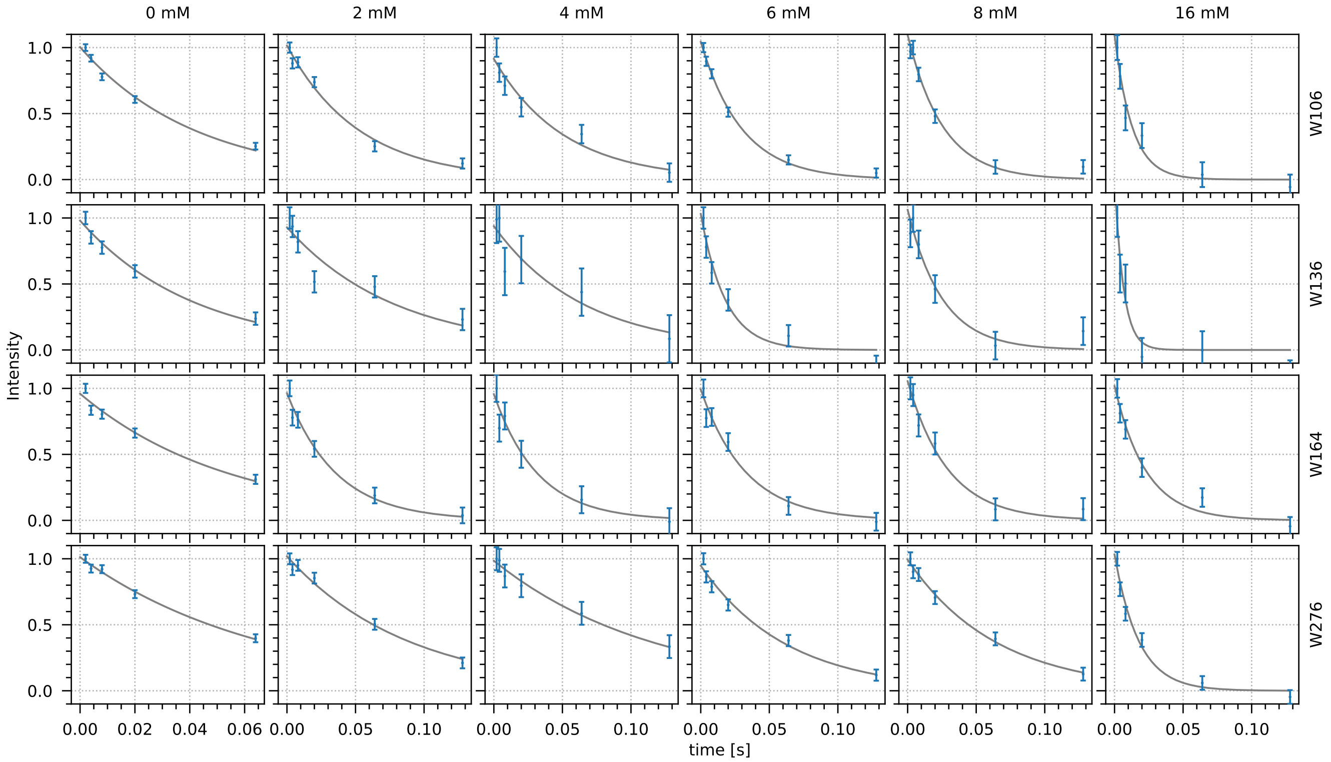

Figure S4: Residue-wise exponential fits of ^{13}C R_2 relaxation experiments (Fig. 1e) at seven different concentrations of Gd(DTPA-BMA). Concentrations and residues are indicated on the top and at the right side, respectively.

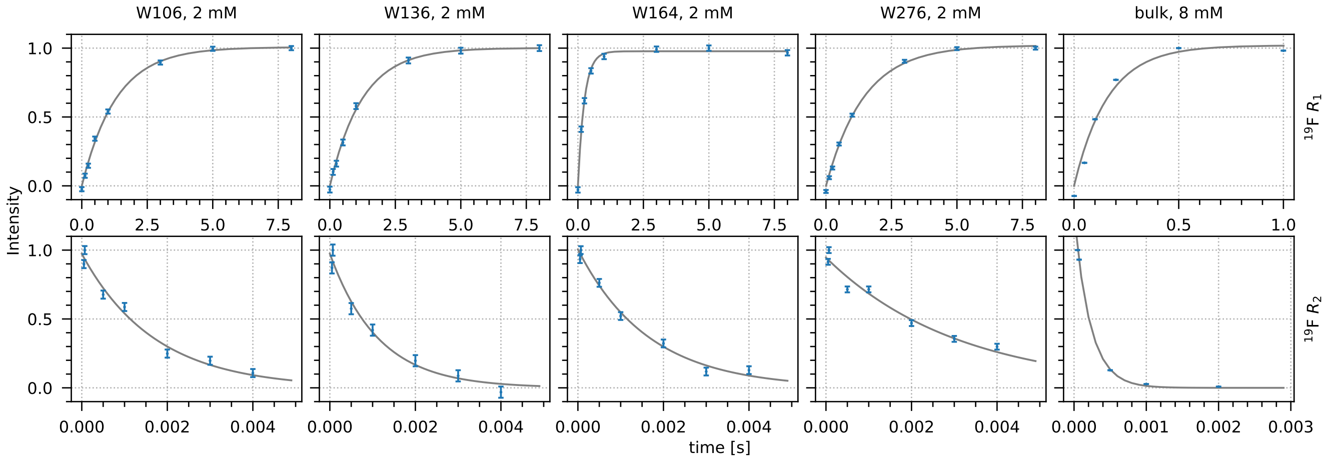

Figure S5: Exponential fits of ^{19}F R_1 (top) and R_2 (bottom) relaxation experiments (Fig. 1b,c) at two different concentrations of Gd(DTPA). The first four columns are residue-wise fits at 2 mM Gd(DTPA). The last column is a fit of the bulk signal at 8 mM Gd(DTPA), as the signals were not resolved due to broadening.

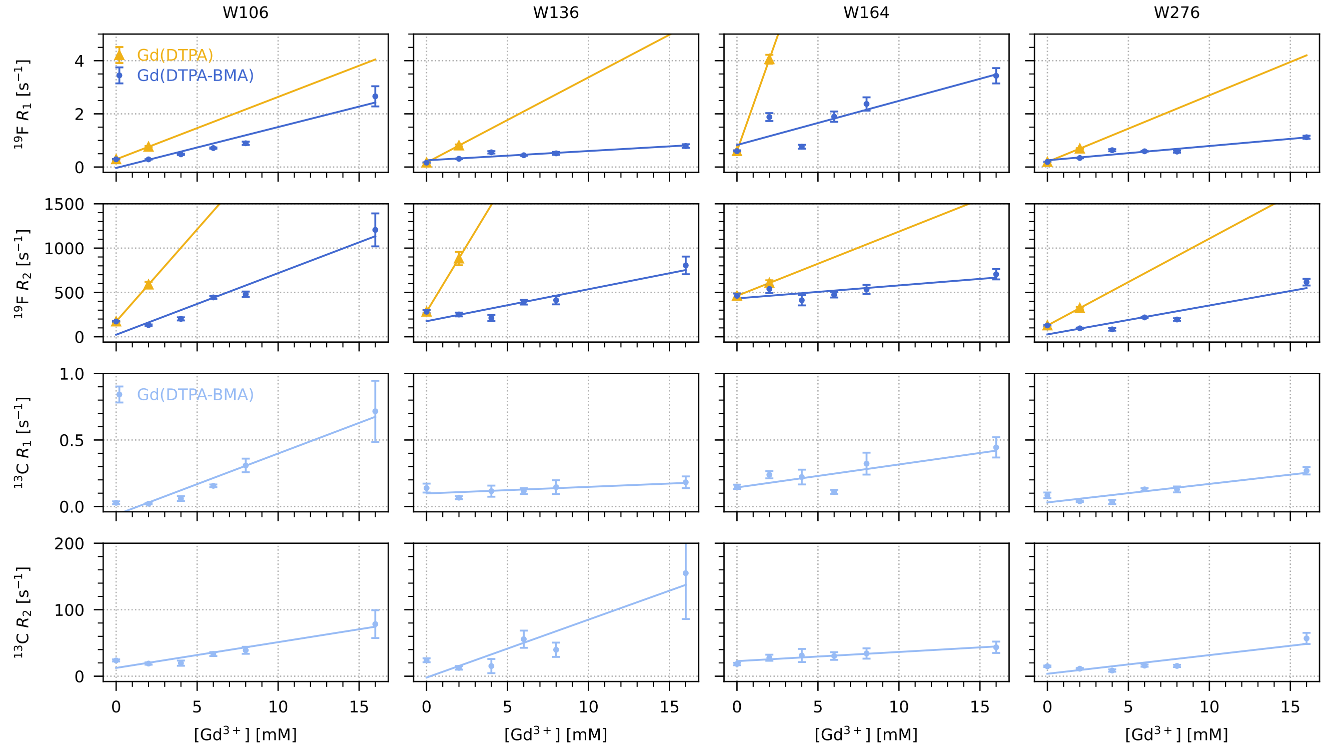

Figure S6: Linear fits of ^{19}F and ^{13}C R_1 and R_2 relaxation rates (see y-axis labels) as a function of the concentration of Gd(DTPA-BMA) and Gd(DTPA) for each tryptophan (indicated on the top).

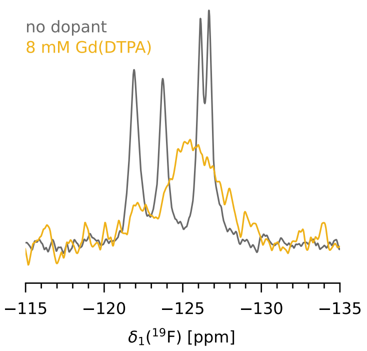

Figure S7: 1D ^{19}F MAS NMR spectrum of 5FC-W-TET without (gray) and with 8 mM Gd(DTPA) (yellow).

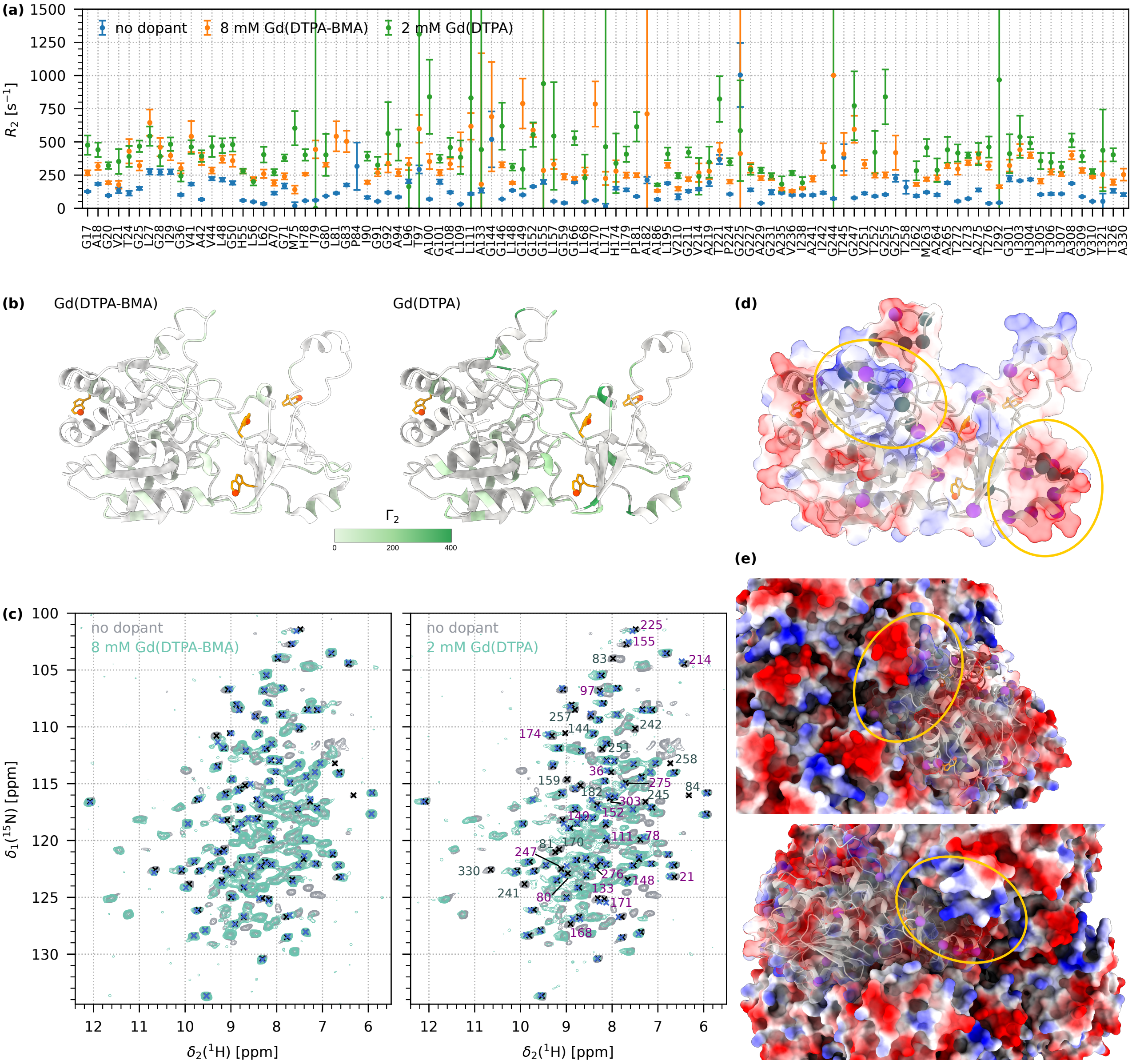

Figure S8: Investigation of specific binding of Gd(DTPA-BMA) and Gd(DTPA) to the surface of TET2. (a) Backbone amide ^{1}H T_2 relaxation of 5FC-W-TET without dopant, with 8 mM Gd(DTPA-BMA), and with 2 mM Gd(DTPA) (note that not all residues are shown). The values for Gd(DTPA) are generally higher than for Gd(DTPA-BMA). (b) ^{1}H Γ_2 for Gd(DTPA-BMA) (left) and Gd(DTPA) (red) obtained from the data in (a) and mapped onto the structure of TET2 (monomer). Residues for which data are missing are white. The tryptophans are shown as orange sticks. (c) ^{1}H–^{15}N MAS NMR spectra of 5FC-W-TET without dopant (gray), with 8 mM Gd(DTPA-BMA) (cyan, left), and with 2 mM Gd(DTPA) (cyan, right). Peaks used in the analysis are marked with a black (no dopant) or blue (with dopant) cross. Labeled peaks in the Gd(DTPA) spectrum (left) are residues with strong chemical shift perturbations (CSP; values larger than 2.5-times the standard deviation over all measured values) (purple) and signals that are broadened beyond detection (dark green). Spectra were recorded at 16.44 T (700 MHz ^{1}H larmor frequency, 1.3 mm HCND probe head), 55.555 kHz MAS, and approximately 309 K. (d) TET2 monomer with the surface colored by the electrostatic potential (positive in blue, negative in red). Residues for which the amide ^{1}H–^{15}N signal is broadened beyond detection in the spectrum with 2 mM Gd(DTPA) are shown as dark green spheres, and residues with strong CSPs are shown as purple spheres. We do not observe a significant accumulation of positive electrostatic potential in the vicinity of the tryptophans. Yellow circles highlight sites that show an accumulation of CSPs and broadened signals. These two sites, in two subunits, are close in space in the dodecamere (see (d)). (e) Same as in (c) but with all subunits shown with the electrostatic potential. The two highlighted sites are adjacent at the subunit interface. The local positive electrostatic potential could lead to specific binding of Gd(DTPA) and explain the observed CSPs and broadened signals. Overall, these data indicate a more site-specific interaction of the Gd(DTPA) complex than of the Gd(DTPA-BMA) complex, which could explain the stronger effect of Gd(DTPA).

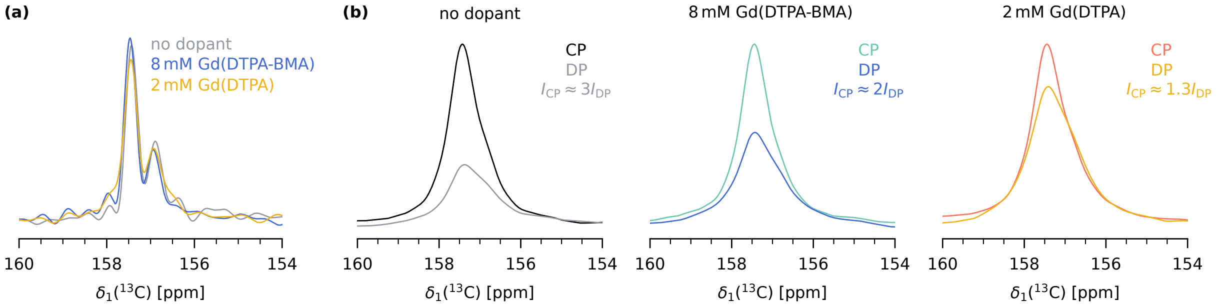

Figure S9: 1D ^{13}C MAS NMR spectra with and without paramagnetic dopants, to understand the effect of the dopants on ^{13}C linewidth and on CP and DP spectra. (a) Direct polarization (DP) spectra of 5FC-W-TET without dopant (gray), with 8 mM Gd(DTPA-BMA) (blue), and with 2 mM Gd(DTPA) (yellow). The ^{13}C line width in the spectrum with Gd(DTPA) is slightly broader than in the other two spectra. The signals of W106, W164, and W276 overlap (left peak). $\tau_{r.d.}$ was set to $5T_1^{bulk}$ of ^{13}C for each sample. Spectra were processed with linear prediction (64 coefficients, 4096 output points) and without apodization. Note that the absolute intensities between samples are not comparable, as the amount of protein inside the rotor is difficult to determine. (b) DP and cross-polarization (CP, $^{19}F-^{13}C$) spectra of 5FC-W-TET without dopant (left), with 8 mM Gd(DTPA-BMA) (middle), and with 2 mM Gd(DTPA) (right). $\tau_{r.d.}$ was set to $5T_1^{bulk}$ of ^{13}C for DP, and ^{19}F for CP spectra for each sample, respectively. Spectra were processed with an exponential apodization function (100 Hz line broadening) and normalized by the square root of the number of scans of CP and DP spectra. Due to the shorter ^{19}F T_1 and the polarization transfer from the more sensitive nucleus, the CP spectra yield more signal. The signal gain for the CP vs. DP spectra is indicated for each panel. For doped samples, the CP efficiency decreases due to faster rotating-frame $R_{1\rho}$ relaxation. However, this reduction of the CP efficiency is over-compensated by the shorter ^{19}F T_1.

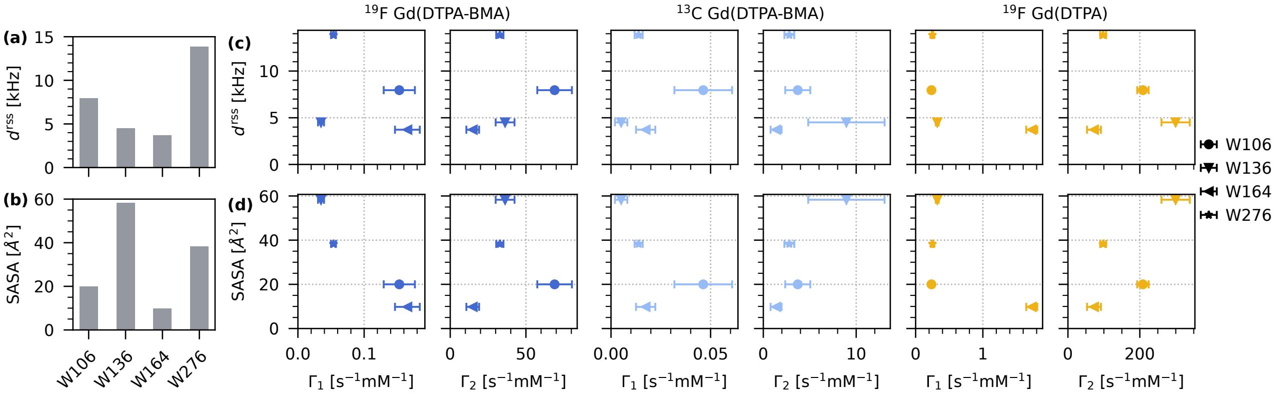

Figure S10: Analysis of tryptophan environments in TET2. (a) Strength of the dipolar coupling network d^{rss} for each tryptophan. All exchangeable protons and all protons of phenylalanines and tyrosines were considered, and distances for the calculations were based on PDB ID 1Y0R (Borissenko and Groll (2005)). (b) Surface accessible surface area (SASA) of tryptophans. The SASA was determined with the gmx sasa function implemented in GROMACS 2023.5 (Abraham et al. (2015)) with a probe radius of 1.4 nm. (c) Correlations between d^{rss} and Γ_1 and Γ_2. (d) Correlations between SASA and Γ_1 and Γ_2.

References

Abraham, M. J., Murtola, T., Schulz, R., Páll, S., Smith, J. C., Hess, B., and Lindahl, E.: GROMACS: High performance molecular simulations through multi-level parallelism from laptops to supercomputers, SoftwareX, 1-2, 19–25, https://doi.org/10.1016/j.softx.2015.06.001, 2015.

Borissenko, L. and Groll, M.: Crystal structure of TET protease reveals complementary protein degradation pathways in prokaryotes, J. Mol. Biol., 346, 1207–1219, https://doi.org/10.1016/j.jmb.2004.12.056, 2005.
